# Genome-wide identification and expression analysis of 3-ketoacyl-CoA synthase gene family in rice (*Oryza sativa* L.) under cadmium stress

**DOI:** 10.3389/fpls.2023.1222288

**Published:** 2023-07-24

**Authors:** Lingwei Yang, Junchao Fang, Jingxin Wang, Suozhen Hui, Liang Zhou, Bo Xu, Yujuan Chen, Yuanyuan Zhang, Changkai Lai, Guiai Jiao, Zhonghua Sheng, Xiangjin Wei, Gaoneng Shao, Lihong Xie, Ling Wang, Ying Chen, Fengli Zhao, Shikai Hu, Peisong Hu, Shaoqing Tang

**Affiliations:** State Key Laboratory of Rice Biology and Breeding, China National Rice Research Institute, Hangzhou, China

**Keywords:** rice, very long-chain fatty acids, β-ketoacyl-CoA synthase, gene family, cadmium stress

## Abstract

3-Ketoacyl-CoA synthase (KCS) is the key rate-limiting enzyme for the synthesis of very long-chain fatty acids (VLCFAs) in plants, which determines the carbon chain length of VLCFAs. However, a comprehensive study of KCSs in *Oryza sativa* has not been reported yet. In this study, we identified 22 *OsKCS* genes in rice, which are unevenly distributed on nine chromosomes. The *OsKCS* gene family is divided into six subclasses. Many *cis*-acting elements related to plant growth, light, hormone, and stress response were enriched in the promoters of *OsKCS* genes. Gene duplication played a crucial role in the expansion of the *OsKCS* gene family and underwent a strong purifying selection. Quantitative Real-time polymerase chain reaction (qRT-PCR) results revealed that most *KCS* genes are constitutively expressed. We also revealed that *KCS* genes responded differently to exogenous cadmium stress in *japonica* and *indica* background, and the *KCS* genes with higher expression in leaves and seeds may have functions under cadmium stress. This study provides a basis for further understanding the functions of *KCS* genes and the biosynthesis of VLCFA in rice.

## Introduction

Very long-chain fatty acids (VLCFAs) are molecules with a hydrocarbon chain containing more than 18 carbon atoms, which are important components of plant cell membrane lipids, cutin wax, and seed storage lipids (Scott et al., 2022). VLCFAs and their derivatives play an important role in plant growth and development, signal transduction, and adverse stress. Disorders in the expression of genes involved in the synthesis of VLCFAs lead to phenotypic consequences, ranging from cell dedifferentiation to embryo lethality (De Bigault Du Granrut and Cacas, 2016; Zhukov and Popov, 2022). VLCFA exists in cell membrane phospholipids and sphingolipids, especially in phosphatidylserine (PS) and phosphatidylethanolamine (PE), participating in intercellular signal transduction to regulate plant growth and development (Boutte and Jaillais, 2020). VLCFAs are precursors of plant cuticle and cutin waxes in epidermal cells, which attach to plant surfaces and are the first line of defense against external stresses (Kunst and Samuels, 2003; Li et al., 2018). VLCFAs can be contained in developing seeds, accounting for up to two-thirds of the total amount of FA. There, they can be in the composition of triacylglycerols (TAGs), playing a key role in seed germination (Cahoon and Li-Beisson, 2020).

The biosynthesis of VLCFA is the elongation of fatty acids from C18 chains to C26–C34 chains via fatty acid elongase (FAE) complex in the endoplasmic reticulum (ER); FAE is composed of four major enzymes such as 3-ketoacyl-CoA synthetase (called as β-ketoacyl-CoA synthetase, KCS), trans-2,3-enoyl CoA reductase (ECR), 3-hydroxacyl-CoA dehydratase (HCD), and 3-ketoacyl-CoA reductase (KCR) (Haslam and Kunst, 2013). In this process, C16:0 or C18:0 or C18:1, the product of *de novo* fatty acid synthesis, is used as a substrate, which is catalyzed through four consecutive reactions in ER, namely, condensation, reduction, dehydration, and secondary reduction; two carbons atoms are added in each cycle (Leonard et al., 2004).

There are two types of non-homologous condensing enzymes involved in fatty acid elongation in organisms: one of these is FAE1-like 3-ketoacyl-CoA synthases (KCS-type enzymes), and the other is ELONGATION DEFECTIVE-LIKE proteins (ELO-LIKEs). ELO-LIKEs are found in many organisms such as humans, plants, and yeasts, whereas KCSs are only found in plants and protists (Stenback et al., 2022). Several studies have been performed on the *KCS* gene family in plants. There are 21 *KCS* genes in *Arabidopsis thaliana* (Joubès et al., 2008), 26 *KCS* genes in *Zea mays* (Campbell et al., 2019), 30 *KCS* genes in *Arachis hypogaea* (Huai et al., 2020), and 58 *KCS* genes in *Gossypium hirsutum* (Xiao et al., 2016). In *Arabidopsis thaliana*, 21 members are divided into four subfamilies according to the amino acid sequence homology: KCS1-like, FDH-like, FAE1-like, and CER6 (Costaglioli et al., 2005), and 21 members are classified into eight subclasses according to their duplication history, genomic organization, protein topology, and 3D modeling (Joubès et al., 2008).

KCS is not only the rate-limiting enzyme in the elongation process of VLCFAs but also has substrate specificity and tissue specificity, which determines the rate of product formation and the carbon chain length of VLCFA. The function of *KCS* gene in *Arabidopsis thaliana* has been thoroughly studied. For example, *KCS18*/*FAE1*, which is specifically expressed in seeds, catalyzes the elongation of C18 to C20 and C22 VLCFAs. *KCS4* is involved in the differential accumulation of polyunsaturated TAGs under stress (Luzarowska et al., 2023). The mutants do not contain C20 and C22 VLCFAs and lead to C18 accumulation (James et al., 1995); *KCS2* and *KCS20* are highly expressed in root endothelium, mainly producing C22 and C24 VLCFAs (Lee et al., 2009); *KCS5* and *KCS6* (*CER6*) play important roles in C24–C28 VLCFAs (Millar et al., 1999), and *KCS3*–*KCS6* module affects wax synthesis (Huang et al., 2023); *KCS9* was the highest expressed in Arabidopsis stem epidermal cells, and the C24 VLCFA of the mutant was significantly reduced (Kim et al., 2013). At present, there are few studies on the function of *KCS* genes in rice. *SD38* is involved in the elongation of C24:0 VLCFA, and the mutant plants are semi-dwarf (Zhang et al., 2022). Two *KCS* genes, *ONI1* and *ONI2*, were specifically expressed in the shoot apical meristem, and mutants with abnormal VLCFA composition resulted in death of plant seedlings (Tsuda et al., 2013). *WSL1* is involved in wax biosynthesis in leaves and leaf sheaths (Yu et al., 2008; Zhou et al., 2021). *WSL4*/*HMS1* is involved in C22:0 VLCFA elongation; its functional deficiency leads to less wax in leaves, shorter plants and fewer tillers, affecting the formation of pollen walls (Gan et al., 2017; Chen et al., 2020).

Cadmium (Cd) is considered as one of the most toxic metals for plant and can cause severe damage both to environment and human. The contamination of Cd in Chinese agricultural soils is quite prevalent, and about a quarter of the soil samples exceed China’s national standard, which are mainly located in the Yangtze River Delta (Cheng et al., 2023). The average of Cd content in brown rice in a survey was slightly higher than milled rice samples and rice Cd content in 35.1% of total 208 cultivars exceed the rice Cd limit (0.2 mg/kg), which were collected in South China (Liu et al., 2023). In many plants, one of the metabolic adaptions to Cd tolerance is related to modifications of fatty acid; the elongation of fatty acid is one way of modifications (Zhukov and Shumskaya, 2020). Similar effect of accumulation of VLCFAs was observed in *Noccaea caerulescens* and tomato plants (Ben Ammar et al., 2008; Zemanova et al., 2015).

Genome-wide identification of gene family provides the basis for further functional analysis. Due to the extensive application of large-scale plant genome sequencing and bioinformatics technology, *KCS* gene families of many species have been identified, but the *KCS* gene family members of rice have not yet been identified. More than 3.5 billion people in the world rely on rice as a staple food and livelihood (Huang et al., 2017). Therefore, in this study, we systematically identified and analyzed the characteristics of rice *KCS* gene family by bioinformatics methods, and the expression levels of *OsKCS* in different tissues were also investigated. Under Cd stress, the expression profiles of *OsKCS* are different in *japonica* and *indica*. This study provided useful information for further investigating the molecular functions of *KCS* genes in rice.

## Materials and methods

### Plant materials and treatment

In this study, the rice variety is Nipponbare and 9311. Seeds were rinsed with distilled water, and then germinated at 28°C under dark conditions. After 48h, seedlings with a root length of approximately 0.5 cm were moved to hydroponic culture boxes (day/night temperatures of 28°C/22°C, light/dark photoperiod of 12h/12h, and light intensity of 18,000 Lx). At the one-leaf stage, the seedlings were treated with nutrient solution. At the two-leaf stage, Cd stress experiments were performed. The CdCl_2_ solutions (20 μmol/L) prepared with nutrient solution were used to simulate Cd stress, and nutrient solution without CdCl_2_ was used as the control. After 3h and 6h of treatment, seedlings were selected for each sample, and quickly stored at -80°C until analysis. The experiment was performed in triplicate.

### Identification of *KCS* genes in rice

In order to identify and characterize *KCS* gene family in rice genome, gff3, proteins, CDS, and genome files were downloaded from Ensembl Plants (http://plants.ensembl.org/Oryza_sativa/Info/Index). Twenty-one identified KCS protein sequences of *Arabidopsis thaliana* were downloaded from Arabidopsis genome database TAIR (https://www.arabidopsis.org/). We performed two methods, which are Basic Local Alignment Search Tool for proteins (BLASTp) and Hidden Markov Models (HMMER) search tool, to identify *KCS* genes in rice genome. The BLASTp (BLAST 2.7.1+) was performed based on protein homologous alignment with default mode using the Arabidopsis KCS protein sequences to obtain the candidate *KCS* genes in rice genome (*E*-value < 10^−10^). The Hidden Markov Model files corresponding to the conserved domain (Accession No.: PF08392 and PF08541) were downloaded from database Pfam (http://pfam.xfam.org/). HMMSEARCH was used to retrieve rice candidate sequences (*E*-value < 10^−20^) containing KCS conserved domains (FAE1_CUT1_RppA and ACP_syn_III_C). Results of these two methods were checked and merged, and the redundancies were manually removed to obtain the candidate *KCS* genes in rice. The candidate sequences were submitted into the NCBI Conserved Domains (https://www.ncbi.nlm.nih.gov/Structure/bwrpsb/bwrpsb.cgi) and SMART (http://smart.embl-heidelberg.de/) to confirm the domains in rice KCS proteins (Letunic et al., 2012).

### Physiochemical properties, alignment and phylogenetic analysis

The *OsKCS* genes’ physical and chemical properties, namely, molecular weight (M.W.), amino acid (aa) length, isoelectric point (pl), instability index, aliphatic index (Ai), and grand average of hydropathicity (GRAVY) were evaluated by using the ExPASY-Prot (http://web.expasy.org/protparam/). The phylogenetic analysis was performed by aligning the KCS protein sequences of rice by MEGA software. The aligned sequences were subjected to neighbor-joining (NJ) tree construction using the MEGA software with 1,000 bootstrap replications and all other parameters were set to default (Hall, 2013).

### Chromosome locations, gene structures, and motif analysis

The chromosome locations and structures of *OsKCS* genes were retrieved from rice genome annotation files, and the conserved motifs of OsKCS protein sequences were predicted by using MEME (MEME 5.1.0) (Bailey et al., 2009). The *OsKCS* gene structures, chromosome locations, and conserved motifs were visualized by TBtools software (Bailey et al., 2009).

### *Cis*-regulatory element analysis of promoters

The 2,000 bp genomic sequences upstream of the transcription start site of *OsKCS* genes were extracted from the genomic DNA sequences. Since the upstream regions of some genes overlap with other genes, the upstream regions of these genes were shortened. The promoter sequences were submitted to the PlantCARE database (https://bioinformatics.psb.ugent.be/webtools/plantcare/html/) to predict *cis*-regulatory elements (Lescot et al., 2002).

### Synteny analysis and Ka/Ks values calculation

The tandem and segmental duplication or whole-genome duplication (WGD) provides new insights into genes development and genome progression. The duplication events of *OsKCS* genes and the syntenic relationships of *KCS* genes between rice and maize were analyzed using MCScanX toolkit (Wang et al., 2012), and *KCS* relationships between the target species were visualized by using Circos (Krzywinski et al., 2009). Nonsynonymous (Ka) and synonymous (Ks) values and the Ka/Ks ratios of gene pairs were calculated by ParaAT 2.0 (Zhang et al., 2012) and KaKs_Calculator (Zhang et al., 2006), Ks value could be used as molecular clock to reckon the time after gene replication event, Ka/Ks ratio has been used to determine the type of gene selection during evolution. Ka/Ks = 1, Ka/Ks > 1, and Ka/Ks < 1 represent natural, positive, and purifying selections, respectively (Hurst, 2002). The divergence time was calculated with the formula: *T* = Ks/*r*; *r* = 6.5 × 10^−9^) (Quraishi et al., 2011).

### Gene expression analysis based on RNA-seq data

To examine the expression pattern of *KCS* genes under Cd stress, three RNA samples of Nipponbare and 9311 were sequenced on the HiSeq 4000 platform (Illumina) for transcriptome analysis by Novogene Technology (Beijing, China) to obtain clean reads. Differentially Expressed Genes (DEGs) were identified by a false discovery rate ≤ 0.05 and an absolute value of the log_2_ ratio ≥ 1. Using the RNA-seq data, the absolute FPKM of the *OsKCS* were obtained, and the R software was used for statistics and visualization. Gene Ontology (GO) and Kyoto Encyclopedia of Genes and Genomes (KEGG) enrichment analysis were performed by R packages: clusterProfiler.

### RNA extraction and qRT–PCR analysis

Total RNA was extracted from different plant tissues using Trizol reagents (Invitrogen, Carlsbad, CA, USA), RNA was reverse transcribed to cDNA using the ReverTra Ace qPCR-RT kit (Toyobo, Osaka, Japan), and qPCR was performed using SYBR Green Real-Time PCR Master Mix (Toyobo).

## Results

### Identification of *KCS* genes in rice

**Figure 1:**
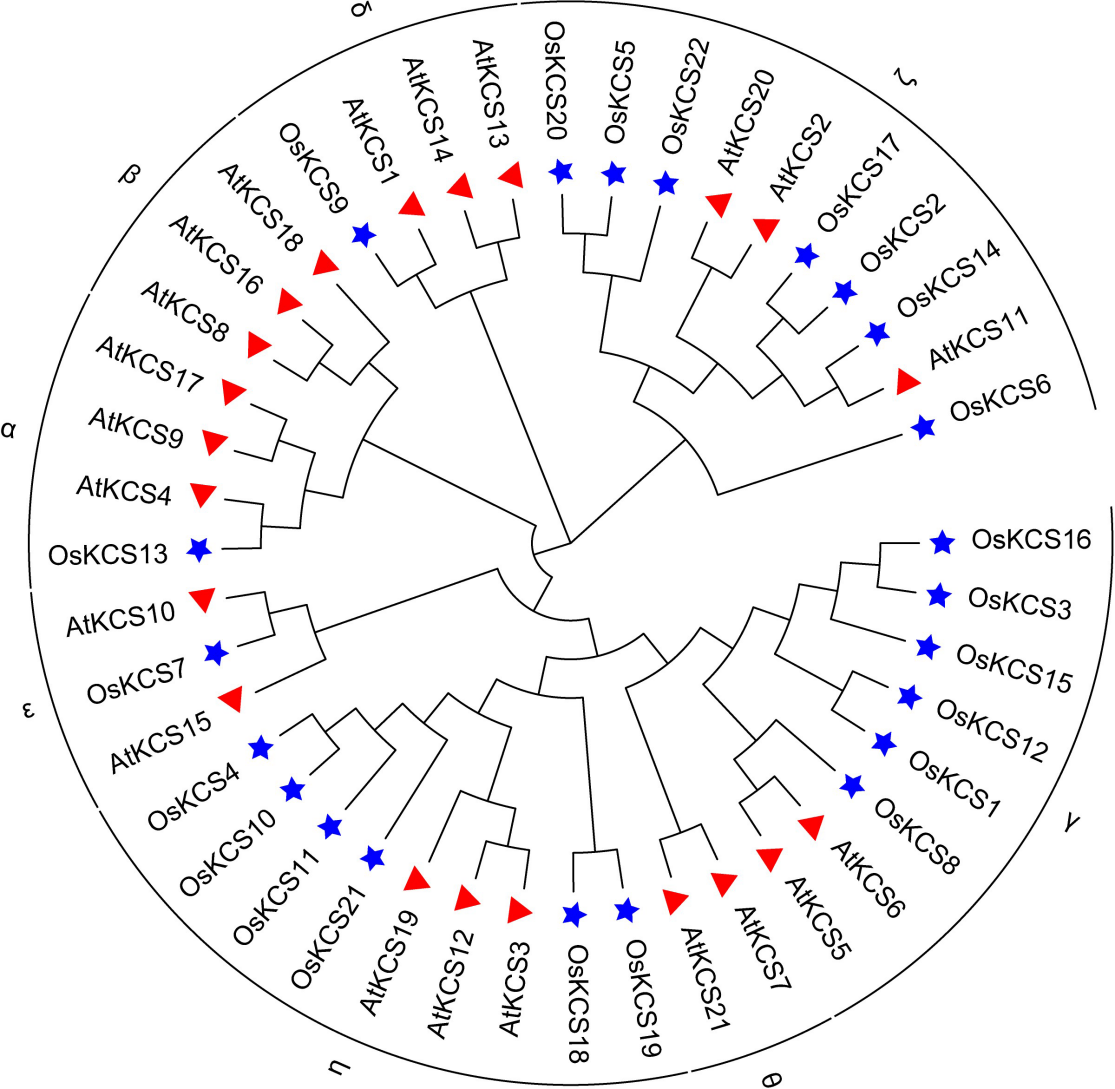
Phylogenetic analysis of the *KCS* genes in *Oryza sativa* (Os) and *Arabidopsis thaliana* (At) by the neighbor-joining method. The KCSs were clustered into eight clades; each member of the KCSs was annotated by (★ for Os) and (▲ for At), respectively.

Twenty-two *KCS* genes in rice genome were identified after removing redundant and repetitive sequences from BLASTp and HMMER results. To explore the phylogenetic relationships of the KCS family, an NJ phylogenetic tree was constructed using the full-length protein sequences of 22 *OsKCSs* and 21 *AtKCSs*. Results demonstrated that KCS proteins were classified into eight clades based on phylogenetic relationship, whereas, KCS proteins in rice were divided into six subclasses: α, γ, δ, ϵ, ζ and η (**Figure 1**). *KCS* genes in rice genome were named *OsKCS1* to *OsKCS22* according to their chromosomal locations. GO and KEGG annotation analysis of the OsKCS genes was performed to further understand the possible roles of OsKCS genes in molecular function, cellular component, and biological process at the molecular levels. GO and KEGG enrichment analysis showed that 22 *OsKCS* genes were all enriched in fatty acid biosynthetic process and involved in fatty acid elongation (**Supplementary Table S1**). Furthermore, the results of physiochemical properties showed that *OsKCS* genes varied in their properties such as the protein length varied from 271 (*OsKCS5*) to 542 aa (*OsKCS7*), as well as the molecular weights ranged from 29.23 to 60.11 kD; the isoelectric point (pI) of OsKCS proteins also varied ranged from 7.67 (*OsKCS10*) to 9.81 (*OsKCS15*), and the protein instability indexes of 11 OsKCS proteins were smaller than 40, indicating that these proteins are stable. The remaining OsKCS proteins’ instability indexes were greater than 40; most KCS proteins are hydrophilic. The *KCS* genes with the highest homology to the *OsKCS* genes and *E*-values were obtained in *Arabidopsis thaliana* (**Table 1**).

**Table 1:** Characterization of the *OsKCS* genes and OsKCS proteins.

Gene	Locus	Amino acids (aa)	Molecular weight (Da)	Isoelectric point (pI)	Instability index	Aliphatic index (Ai)	GRAVY	*Arabidopsis* homologous gene		*E*-value
								Accession no.	Name	
*OsKCS1*	LOC_Os01g34560	478	52280.7	8.16	35.55	98.18	0.113	AT1G68530	*AtKCS6*	1.49E-171
*OsKCS2*	LOC_Os02g11070	519	57689.1	8.96	43.58	93.93	−0.045	AT2G26640	*AtKCS11*	0.00E+00
*OsKCS3*	LOC_Os02g49920	501	55267.5	9.42	43.75	93.73	0.022	AT1G68530	*AtKCS6*	0.00E+00
*OsKCS4*	LOC_Os02g56860	463	50765.8	8.85	44.75	91.77	−0.028	AT2G28630	*AtKCS12*	3.51E-149
*OsKCS5*	LOC_Os03g06700	271	29227.4	9.32	45.95	92.62	0.13	AT2G26640	*AtKCS11*	2.09E-52
*OsKCS6*	LOC_Os03g06705	307	33803.3	9.31	40.67	83.88	−0.167	AT2G26640	*AtKCS12*	3.12E-123
*OsKCS7*	LOC_Os03g08360	542	60108.5	9.36	48.08	88.58	−0.061	AT2G26250	*AtKCS10*	0.00E+00
*OsKCS8*	LOC_Os03g12030	494	55786.5	9.45	43.92	94.74	−0.04	AT1G68530	*AtKCS6*	0.00E+00
*OsKCS9*	LOC_Os03g14170	532	59315.0	9.1	32.72	86.56	−0.102	AT1G01120	*AtKCS1*	0.00E+00
*OsKCS10*	LOC_Os03g26530	467	50907.7	7.67	38.66	90.54	−0.06	AT2G28630	*AtKCS12*	3.23E-176
*OsKCS11*	LOC_Os03g26620	472	52178.6	8.65	46.95	89.56	−0.106	AT2G28630	*AtKCS12*	5.85E-145
*OsKCS12*	LOC_Os04g02640	429	47007.9	9.33	38.36	98.41	0.128	AT1G68530	*AtKCS6*	3.62E-99
*OsKCS13*	LOC_Os05g49290	514	57427.1	9.23	36.45	95.04	−0.037	AT1G19440	*AtKCS4*	0.00E+00
*OsKCS14*	LOC_Os05g49900	520	58046.7	9.41	37.91	92.46	−0.1	AT2G26640	*AtKCS11*	0.00E+00
*OsKCS15*	LOC_Os06g14810	527	57414.2	9.81	38.98	87.27	−0.134	AT1G68530	*AtKCS6*	0.00E+00
*OsKCS16*	LOC_Os06g15170	494	54249.2	8.47	38.17	95.06	0.148	AT1G68530	*AtKCS6*	0.00E+00
*OsKCS17*	LOC_Os06g39750	519	58011.8	9.25	39.76	92.62	−0.088	AT2G26640	*AtKCS11*	0.00E+00
*OsKCS18*	LOC_Os09g19650	482	52636.1	9.78	47.99	85.46	0.034	AT4G34510	*AtKCS17*	8.63E-127
*OsKCS19*	LOC_Os09g34930	439	47175.6	8.94	28.85	82.73	−0.049	AT1G19440	*AtKCS4*	3.40E-97
*OsKCS20*	LOC_Os10g28060	523	56867.1	9.63	32.05	95.37	0.048	AT1G04220	*AtKCS2*	0.00E+00
*OsKCS21*	LOC_Os10g33370	465	51550.2	9.5	47.51	88.11	−0.131	AT2G28630	*AtKCS12*	1.56E-157
*OsKCS22*	LOC_Os11g37900	432	47165.7	9.15	42.25	86.34	−0.014	AT1G04220	*AtKCS2*	0.00E+00

### Gene structures and conserved motif analysis of *OsKCS*

**Figure 2:**
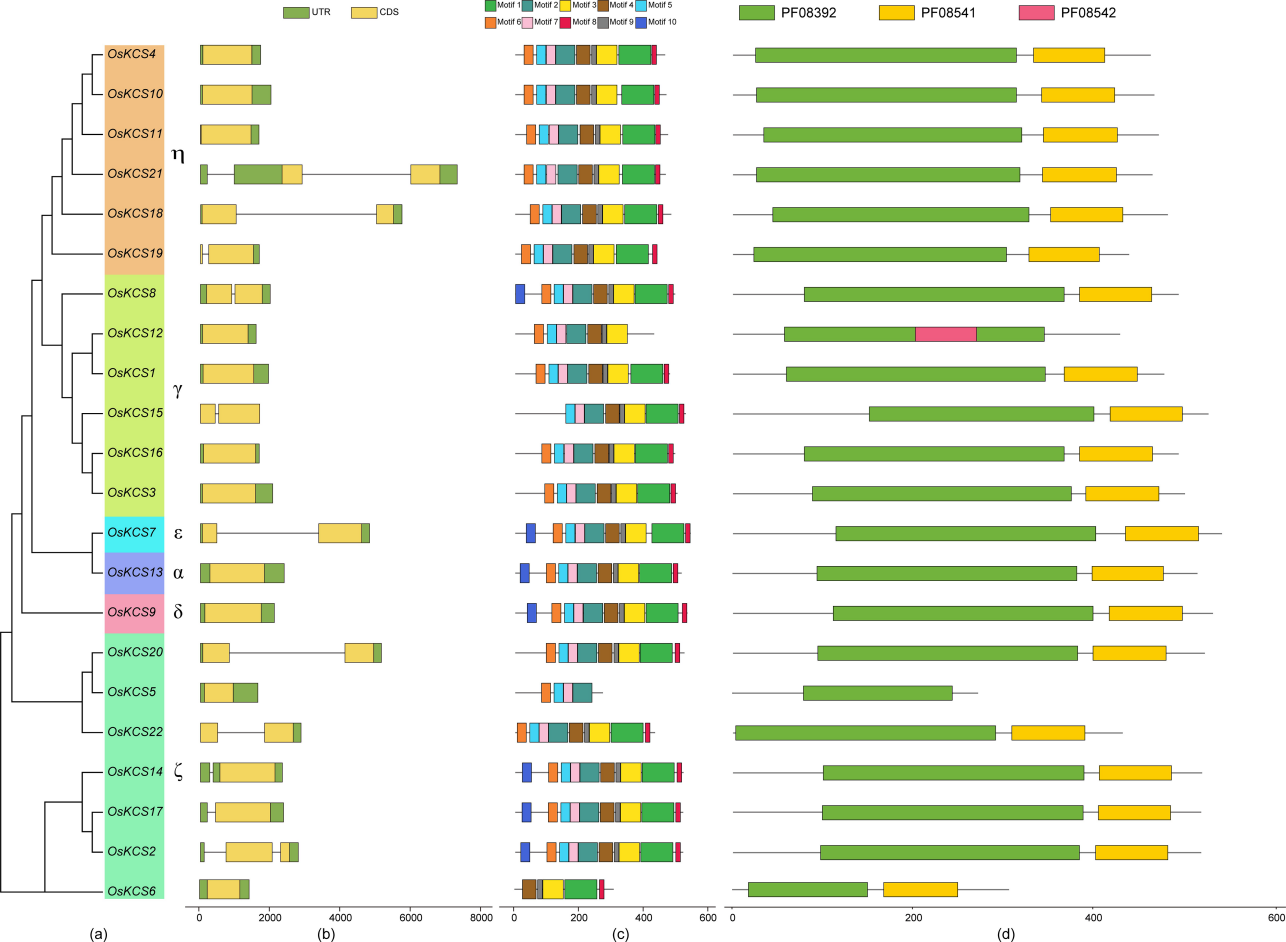
The unrooted phylogenetic tree, conserved motifs, and gene structure of *OsKCS*. **(A)** The neighbor-joining tree on the left composed of 22 KCS proteins from rice. **(B)** *OsKCS* genes structures: yellow color indicates the exons, the green color shows the untranslated 5′ and 3′ regions. **(C)** Conserved motifs were represented via boxes and different colors represents different motifs. **(D)** The function conserved domains of *OsKCS* genes. PF08392: FAE1_CUT1_RppA; PF08541: ACP_syn_III_C; PF08542: ACP_syn_III.

OsKCS proteins were classified into four main groups (KCS1-like, FAE1-like, CER6-like, and FDH-like) (**Figure 2A**). Most of the *OsKCS* gene family members contained 1–2 exons, of which 11 *OsKCS* genes did not contain introns (**Figure 2B**). With the exception of *OsKCS5*, which has only four motifs, most of the conserved motifs of the *OsKCS* gene family members have the same types, numbers, and orders (**Figure 2C**). CDD and SMART search tools for domains verification was used and found that OsKCS proteins contained two domains such as FAE1_CUT1_RppA [(PF08392) FAE1/Type III polyketide synthase-like protein domain] and ACP_syn_III_C [(PF08541) 3-Oxoacyl-acyl-carrier protein (ACP) synthase III C terminal domain], which were main conserved domains in KCS proteins (**Figure 2D**), almost all genes contain these two domains except for *OsKCS12*.

### *Cis*-regulatory element analysis of *OsKCS*

**Figure 3:**
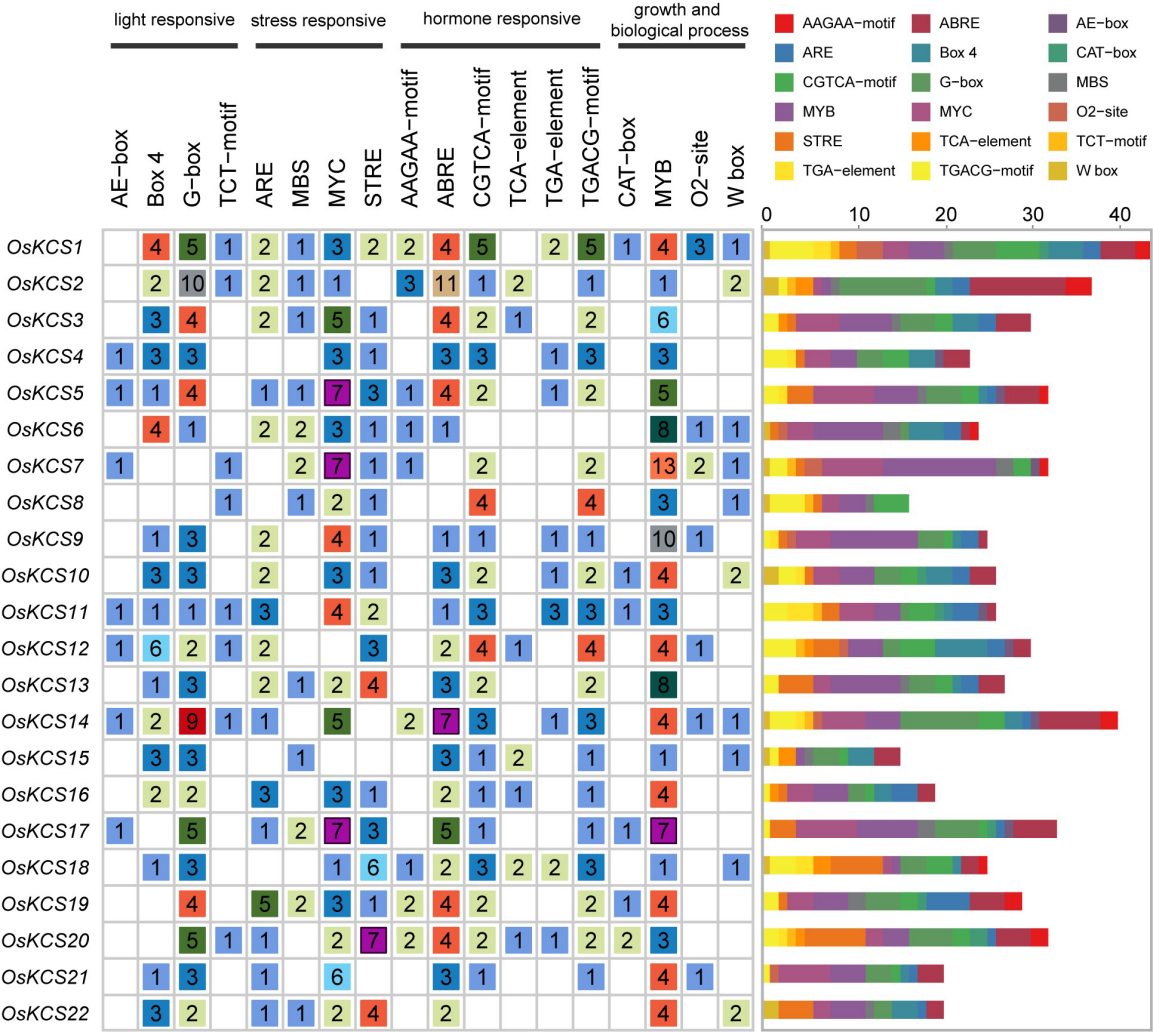
*Cis*-elements found in the promoter region of *OsKCS* genes. (Left): The number and function classification of *cis*-acting element in each *OsKCS* genes. (Right): Distribution of 18 identified *cis*-acting elements in each *OsKCS*; elements are represented by the boxes in different colors.

A total of 18 *cis*-regulatory elements were predicted in the upstream 2,000 bp from the transcription start sites of *OsKCSs* (**Figure 3**), which were widely involved in the growth biological process, hormonal responsiveness, light responsiveness, and stress response. A list of genomic location and names/annotations of 22 *OsKCS*s and the respective upstream genes will be provided to know whether any of the *KCS*-upstream gene blocks are paralogous (**Supplementary Table S2**). Among them, MYB transcription factors involved in plant biological process were identified in all *OsKCSs*. Four types of hormone-responsive elements were also found, namely, auxin-responsive elements (TGA-elements) in nine *OsKCSs*, MeJA-responsive elements (CGTCA-motif and TGACG-motif) in 20 *OsKCSs*, salicylic acid–responsive elements (TCA-element) in seven *OsKCSs*, and ABA-responsive elements (ABRE) in 20 *OsKCSs*. Furthermore, there were meristem expression-related elements (CAT-box) in seven *OsKCSs* and gliadin metabolism-related elements in seven *OsKCSs*. The results of *cis*-regulatory element analysis indicate that *OsKCS* may be expressed in different growth environments, hormones, and stress treatments. However, many motifs have not been functionally verified, and whether these motifs confer unique functions on *OsKCS* remains to be further studied.

### Chromosome distribution and synteny relationship of *OsKCS*

**Figure 4:**
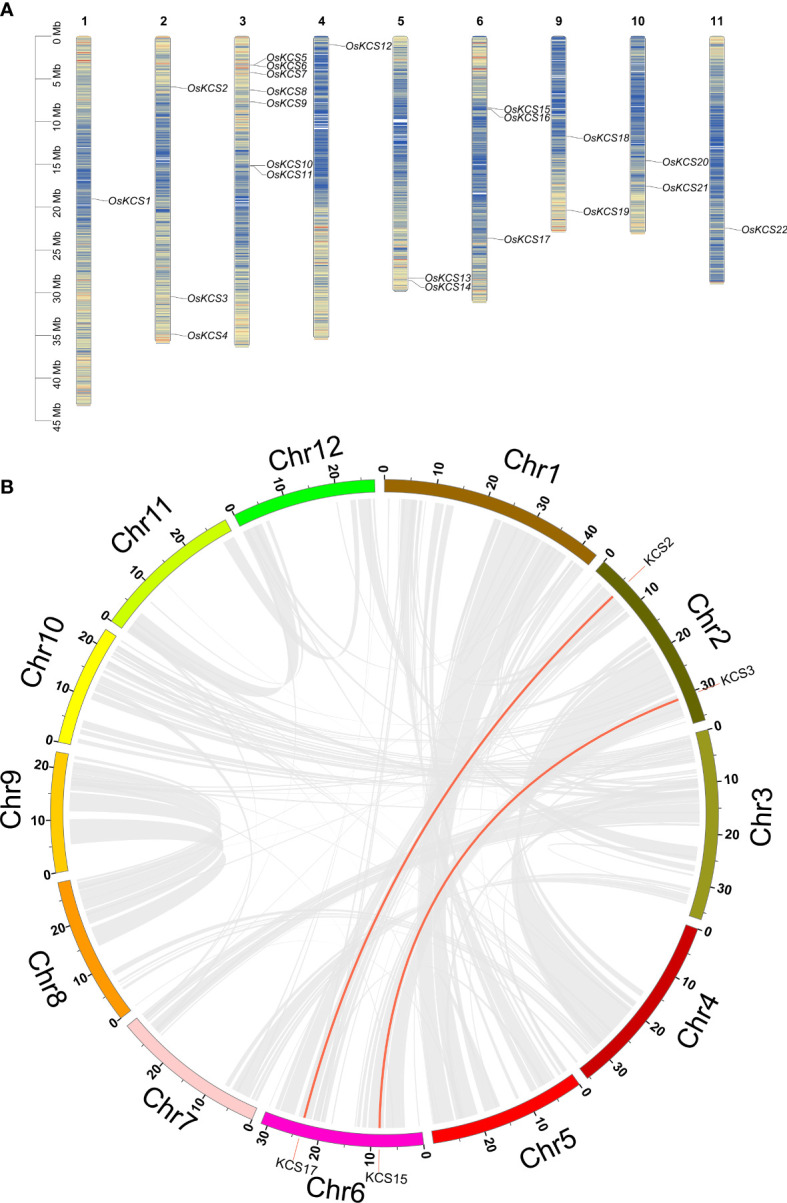
Chromosome distribution and synteny relationship of *OsKCS* gene family. **(A)** Chromosome location of 22 *OsKCS* genes in rice. **(B)** Circle map of the duplication gene pairs of the *OsKCS* genes. The background gray lines show all the syntenic blocks in the rice genome, and the red lines show the segmental duplication link regions among *OsKCS* genes.

**Figure 5:**
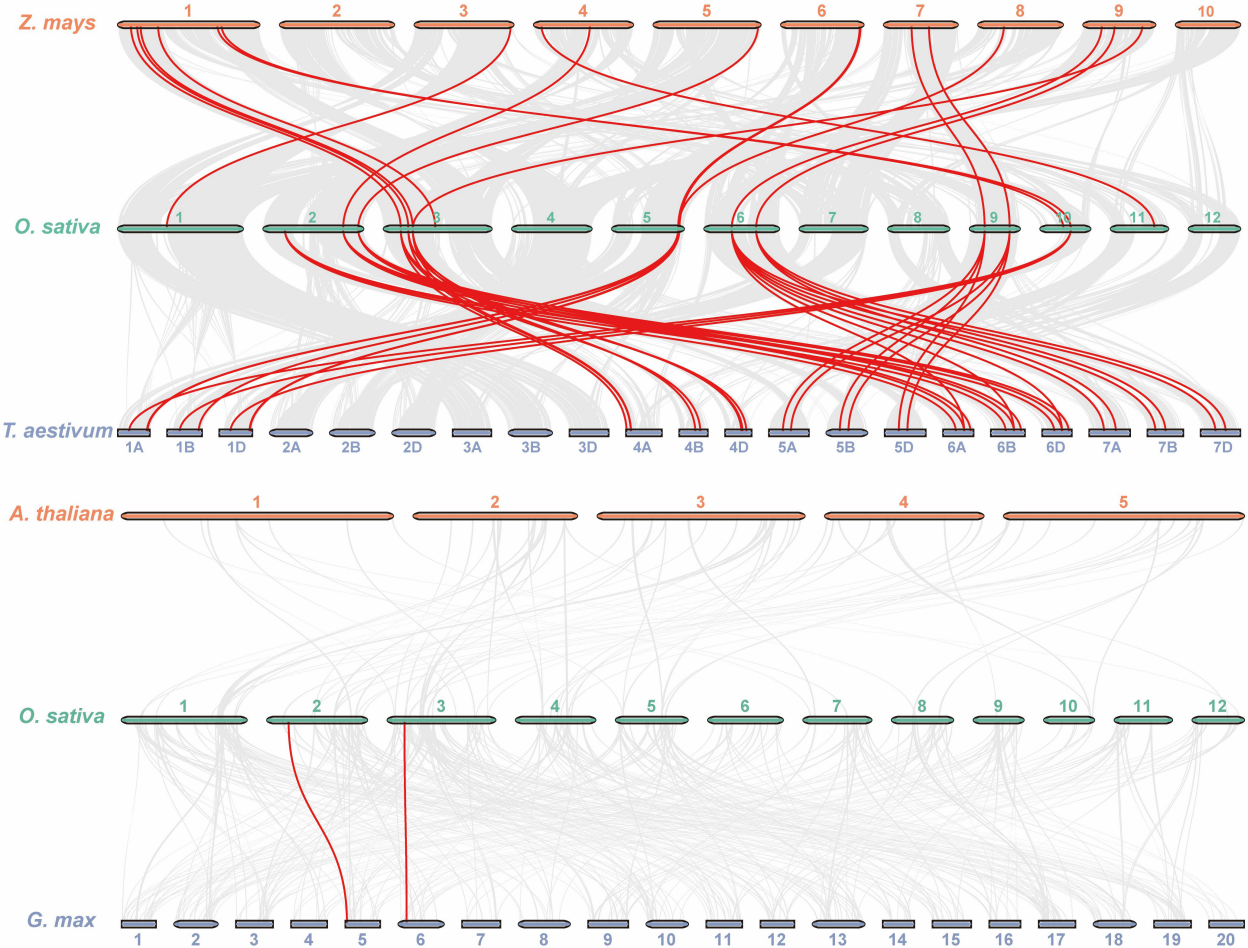
Synteny analysis of *KCS* genes between rice and four representative plant species. The red lines highlight the syntenic *KCS* gene pairs; the gray lines in the background represent the collinear blocks in rice that are orthologous to the other plant genomes.

Except for chromosomes 7, 8, and 12, all of the *OsKCS* genes were unevenly distributed on nine of 12 chromosomes (**Figure 4A**). The largest number of *OsKCS* genes (*OsKCS5*–*OsKCS11*) appeared on chromosome 3, followed by chromosomes 2 and 6 (three genes each), chromosomes 5, 9, and 10 (two genes each). Synteny analysis was used to understand the evolution and expansion mechanism of *OsKCS* gene family in the rice genome and the genomes of other species. The results of gene duplication analysis indicated that there were only two *OsKCS* gene pairs (*OsKCS2*/*OsKCS17* and *OsKCS3*/*OsKCS15*), both were segmentally duplicated on chromosomes 2 and 6 (**Figure 4B**). There were no tandem duplications in tight regions on chromosomes 3 and 6. Ka/Ks value is used to evaluate the evolution of coding sequences and determine the type of selection pressure after duplication. The Ka/Ks values of the two gene pairs were smaller than 0.05, indicating that these genes had gone through purifying selection (**Supplementary Table S3**). The results of the divergence time indicated that the duplication process between the segmental *OsKCS* genes was estimated to be 6 million years ago, and the evolutionary mechanism of *OsKCS* was conserved during evolution. In order to further understand the evolutionary origins and orthologous relationship of *KCS* gene family, the synteny analysis was performed among four representative plant species (two dicots: *Arabidopsis* and *Glycine max*; two monocots: *Zea mays* and *Triticum aestivum*) (**Figure 5**). Sixteen and 40 orthologous pairs were found between *Zea mays* and *Triticum aestivum*, respectively. Two and 0 orthologous pairs were found between *Glycine max* and *Arabidopsis*, respectively. The huge differences in homologous gene pairs between dicots and monocots suggested that *KCS* genes may be formed after the differentiation of monocots and dicots.

### Expression characteristics of *OsKCS*

**Figure 6:**
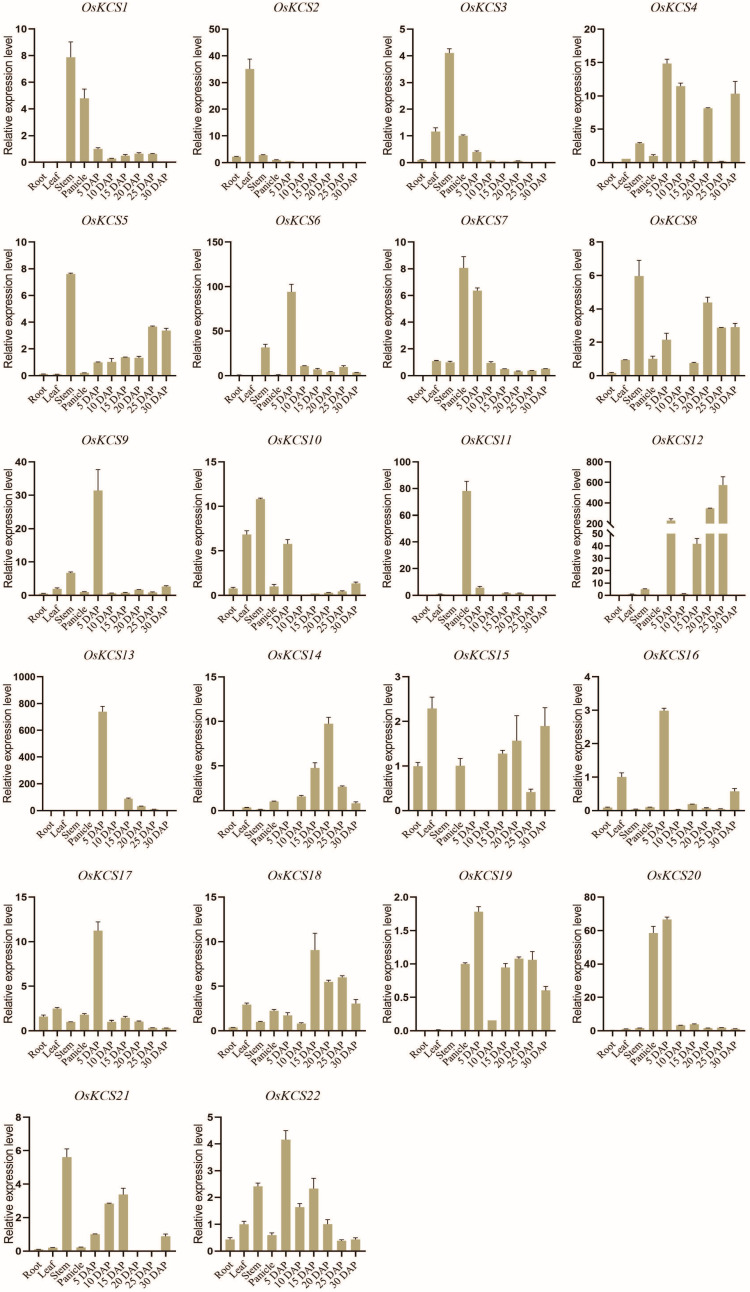
Expression of 22 *OsKCS* genes in different rice tissues using qRT-PCR.

To investigate the rice *KCS* genes expression patterns in different tissues of rice plants, we analyzed rice transcript expression (RNA-seq data) in four different tissues; this included the expression in the root, leaf, panicle, and mature seed (**Supplementary Figure S1**). In order to further investigate the functions of *OsKCS* genes involved in different developing stages, qRT-PCR was performed on all *KCS* gene members (**Figure 6**). These results indicated that the predictions of expression profiles of most *KCS* genes were consistent with the qPCR data. *KCS* genes were specifically expressed in leaves (two genes), stem (six genes), panicle (three genes), and seeds (11 genes), respectively, and most of the *KCS* genes (*OsKCS4*, *OsKCS6*, *OsKCS9*, *OsKCS13*, *OsKCS16*, *OsKCS17*, and *OsKCS22*) expressed in seeds were highly expressed in the early stage of growth and development, suggesting that these *OsKCS* genes played distinct roles during the development of grain.

### Expression patterns of *OsKCS* under cadmium stress based on RNA-seq

**Figure 7:**
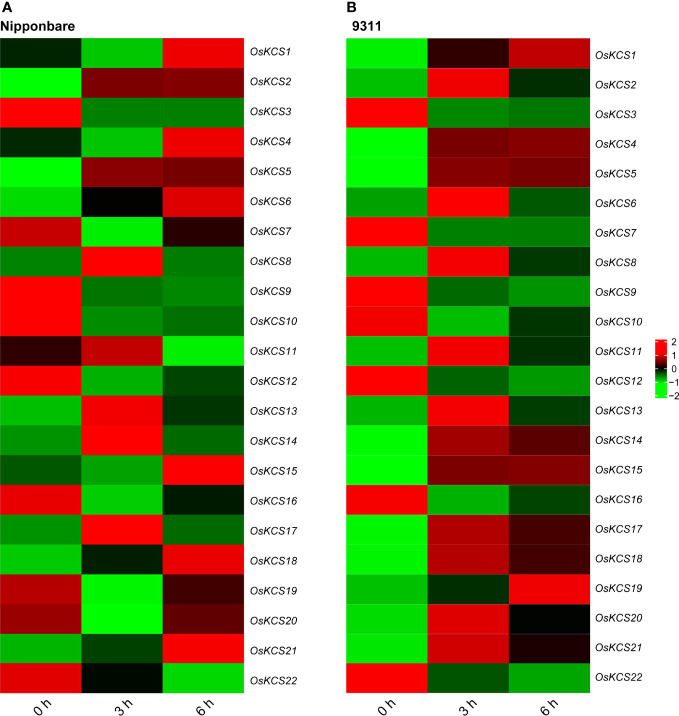
Expression patterns of *OsKCS* genes in shoots under exogenous cadmium treatment. **(A, B)** Heatmaps of gene expression levels treated with 20 μM Cd in *japonica* Nipponbare and *indica* 9311.

Based on the analysis of *cis*-elements in the promoter of *OsKCS* genes, all of these genes were hypothesized to respond to stress. Cadmium (Cd) is considered as one of the most toxic metals for plant. In order to analyze *OsKCS* involved in the response to Cd stress, we analyzed the RNA-seq data to evaluate the response of 22 *OsKCS* to Cd treatment in *indica* and *japonica*. All *OsKCSs* were differently expressed under Cd stress. In *japonica* Nipponbare background, after 3h treatment, the expression of 12 genes was significantly down-regulated, and the expression of these genes was different between 3h and 6h treatment (**Figure 7**). For example, *OsKCS8*, *OsKCS13*, *OsKCS14*, and *OsKCS17* were up-regulated after 3h treatment, the expression of these genes decreased gradually in 6h but was still higher than 0h (control). In *indica* 9311 background, 15 genes were up-regulated and seven genes were down-regulated continuously with the extension of treatment, seven genes were down-regulated and two genes were up-regulated under 6h of Cd treatment, 13 genes were up-regulated under 3h of Cd treatment. Whether in *indica* or *japonica*, *OsKCS2*, *OsKCS5*, *OsKCS6*, *OsKCS8*, *OsKCS13*, *OsKCS17*, *OsKCS18*, and *OsKCS21* were up-regulated gradually with the extension of treatment time. The expression of *OsKCS19* and *OsKCS20* was opposite in *indica* and *japonica* after Cd treatment. The results showed that most *OsKCSs* were sensitive to the Cd.

## Discussion

The β-ketoacyl-CoA synthase (KCS) family plays an important role in regulating plant growth and development and resisting abiotic stress (De Bigault Du Granrut and Cacas, 2016; Batsale et al., 2021). The identification and analysis of the *OsKCS* gene family at the genome level could provide a theoretical basis for functional characterization. In the current study, a total of 22 *OsKCS* genes were identified in the rice genome; the number of identified *KCS* genes in the rice genome was slightly higher compared with *Arabidopsis thaliana* (21), but smaller than that of *Zea mays* (26) and *Brassica campestris* (46), which may be due to the differences in genome size and the time when the duplication event occurred. The specific domain (FAE1_CUT1_RppA) was conserved in OsKCS proteins and all of *KCS* genes have this domain (Huai et al., 2020; Tong et al., 2021; Rizwan et al., 2022), indicating that 22 KCS proteins of rice containing the active motif involved in the elongation of VLCFAs.

Using sequence alignment and phylogenetic tree construction, the grouping and evolutionary relationships of the rice *KCS* gene family were determined. The phylogenetic tree categorized the rice *KCS* gene family into six subclasses. The grouping and clustering of KCS proteins were caused by differences in protein sequences. Twenty-one KCSs in *Arabidopsis thaliana* are classified into eight subclasses: α, β, γ, δ, ϵ, ζ, η and θ (Joubès et al., 2008). The KCSs in subclasses α, β, γ, δ and ϵ are known to possess catalytic activity, because they could display activity in various heterologous yeast expression systems (Blacklock and Jaworski, 2006; Paul et al., 2006). However, the *KCS3* in *Arabidopsis thaliana* belongs to subclass θ, and it plays a negative regulator of wax metabolism by reducing the enzymatic activity of KCS6, a key KCS involved in wax production (Huang et al., 2023). KCSs in *Oryza sativa* were not classified into β and θ; it is possible that most of the KCS in rice have catalytic activity, which needs further experimental verification. There were some differences in the conserved motifs of OsKCS proteins among different subclasses. The differences in the distribution of these conserved motifs indicated the different functions of *OsKCS* genes. The differences in gene structure might play a role in gene evolution. The intron–exon structure of the 22 *OsKCS* genes is different, and the differences in structure will lead to different functions (Xu et al., 2012).

*Cis*-regulatory elements play an essential role in gene’s spatiotemporal expression, and further in regulating plant growth and development, as well as in coordination and adaptation to the environment (Priest et al., 2009). The *cis*-regulatory element analysis in *OsKCS* genes was performed and found that MYB transcription factor binding sites existed in the promoter regions of its members. Previous studies have also shown that MYB transcription factors may regulate *KCS* and further regulate VLCFA and plant function in growth and development (Raffaele et al., 2008; Xu et al., 2022; Yang et al., 2023).

The *KCS* gene family in plants has formed a huge gene family through duplication and has accumulated many mutations after a long evolution, leading to the differentiation of gene functions. Some studies believed that the *KCS* genes in plants are the results of large-scale duplication events such as WGDs or segmental chromosomal duplications (Guo et al., 2016). The rice genome did not contain any tandem *KCS* genes but contained four segmental duplication genes. The large-scale duplication events of *KCS* genes in rice are smaller than that in *Arabidopsis* and *Passiflora* (Rizwan et al., 2022). Therefore, the dominating duplication mode of *KCS* genes in rice appeared to be WGDs or segmental duplications. The number of orthologous pairs of *KCS* genes between *Grapevine* and *Arabidopsis* is more than that between *Oryza* and *Arabidopsis*(Zheng et al., 2023).

Measurements of the adverse effects of heavy metal on plants generally are related with seed germination, root length, and morphologic growth. A study showed that the fatty acid composition (C18) of leaves was also correlated with heavy metals accumulation in soils, and the fatty acid composition of leaves could be used as an indicator of the adverse effects of heavy metals on plants. (Verdoni et al., 2001). Under heavy metal stress, *HMA3* overexpressing transgenic plants displayed a higher quantity of fatty acids (C18-20)s in seed (Park et al., 2015). These studies indicated that the fatty acid composition was altered after metal stress. VLCFAs are synthesized in the ER through C18 via FAE complex. The expression levels of eight genes (*OsKCS2*, *OsKCS5*, *OsKCS6*, *OsKCS8*, *OsKCS13*, *OsKCS17*, *OsKCS18*, and *OsKCS21*) were gradually up-regulated with the extension of treatment time and were higher in leaves and seeds compared with other tissues. The *OsKCS* with high expression in leaves and seeds might play a role in cadmium stress.

## Conclusions

In this study, a total of 22 *OsKCS* genes were identified in the rice genome. OsKCS is divided into six subclasses, and the physiochemical features of KCS protein were different. *OsKCS* gene family has undergone purification selection. The qRT-PCR based expression suggested that *OsKCS* genes are specifically expressed in different tissues. The *KCS* genes in *indica* and *japonica* with high expression in leaves and seeds might play roles under Cd stress. These findings provide a basis for further studies on the functions of *KCS* genes in rice. In future studies, we will further explore the role of *OsKCS* genes in VLCFA.

## Data availability statement

The original contributions presented in the study are included in the article/**Supplementary Material**. Further inquiries can be directed to the corresponding authors.

## Author contributions

Conceptualization, LY and SKH; methodology, JF, JW, LY, and SZH; validation, LY, JF, LZ, BX, and YZ; formal analysis, JW, LY and YJC; resources, ZS, XW, YC, FZ, and GS; data curation, GS, LW, and LX; writing—original draft preparation, LY and JF; writing—review and editing, LY, JW, and SKH; supervision, SKH, PH, and ST. All authors contributed to the article and approved the submitted version.

## Supplementary material

Supplementary Figure 1 Expression pattern of *OsKCS* genes in panicle, seed, root, leaf in RNA-seq.

Supplementary Table 1 Enrichment analysis of *OsKCS* genes.
