# Supplementary figures and images for: Genome-wide identification and expression analysis of 3-ketoacyl-CoA synthase gene family in rice (Oryza sativa L.) under cadmium stress
Source: Front Plant Sci. 2023 Jul 24;14:1222288. doi: 10.3389/fpls.2023.1222288 (PMC10406525; doi:10.3389/fpls.2023.1222288)

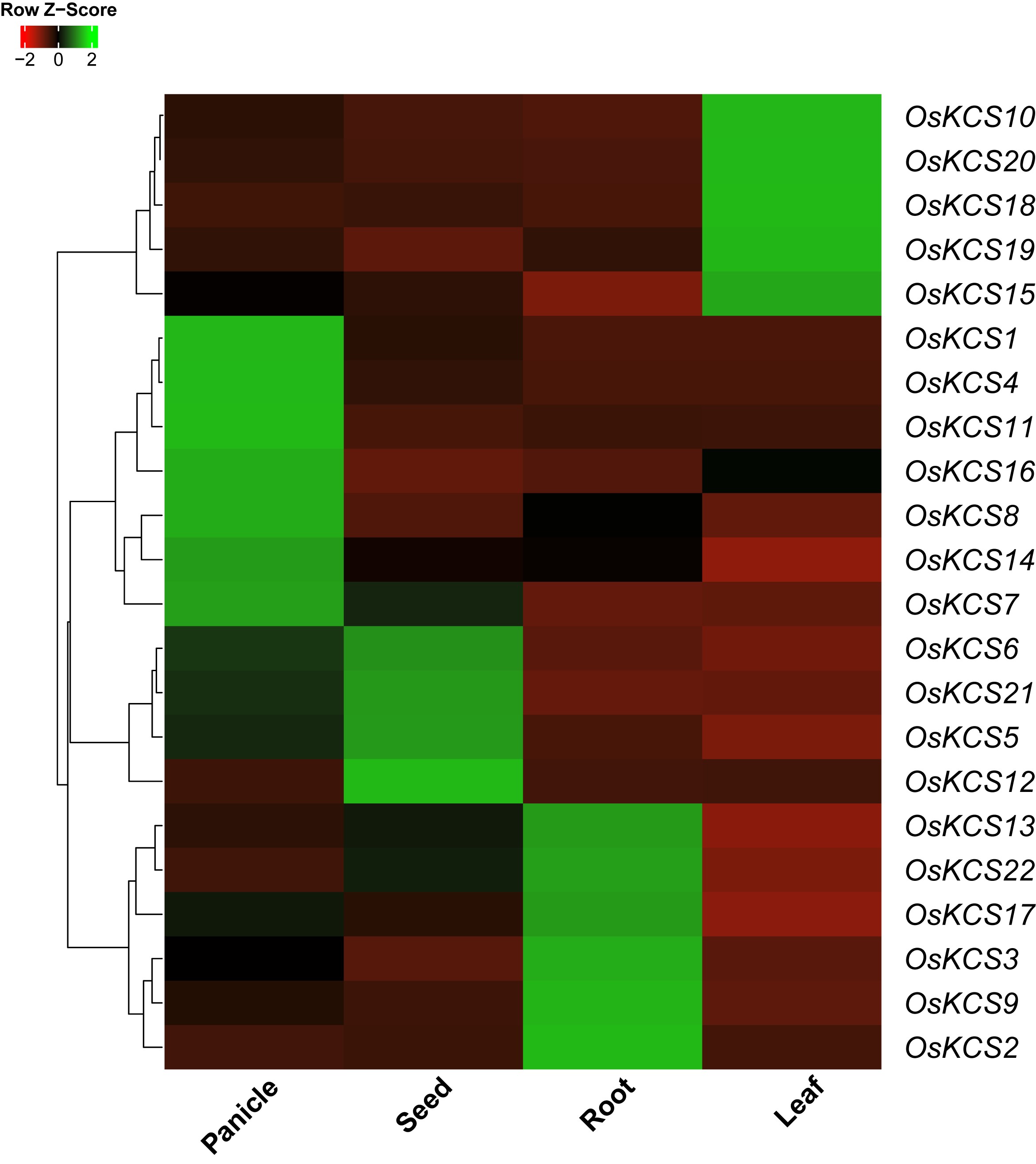

Supplement: Supplementary Figure 1 — Expression pattern of OsKCS genes in panicle, seed, root, leaf in RNA-seq. [file Image_1.jpeg]
